# Caspase-3 and Survivin Expression in Primary Atypical and Malignant Meningiomas

**DOI:** 10.1155/2013/626290

**Published:** 2013-12-19

**Authors:** Andrej Vranic

**Affiliations:** ^1^Department of Neurosurgery, University Medical Center Ljubljana, Zaloska 7, 1525 Ljubljana, Slovenia; ^2^Génomique Fonctionnelle des Tumeurs Solides, Unité INSERM U674, 75010 Paris, France

## Abstract

*Objective*. Information about possible prognostic factors of the survival of patients with atypical and malignant meningiomas (AMM) is sparse. The aim of our study was to evaluate prognostic significance of apoptotic marker caspase-3 and apoptotic inhibitor survivin in a series of primary AMM. *Methods*. 86 AMM (76 atypical and 10 malignant) were analyzed. Caspase-3 and survivin expression was evaluated immunohistochemically. The correlation between caspase-3, survivin, and other possible factors of meningioma recurrence was evaluated. Uni- and multivariate recurrence-free survival (RFS) and overall survival (OS) analyses were performed. *Results*. The intensity of caspase-3 expression correlated with the tumor grade (*P* = 0.004), the proliferation index (*P* = 0.019), and the mitotic count (*P* = 0.013). Survivin tended to be more expressed in female patients (*P* = 0.072). Survivin expression was stronger in malignant compared to atypical meningiomas, however, the difference was not statistically important (*P* = 0.491). Neither survivin nor caspase-3 expression significantly predicted OS or RFS in patients with AMM. *Conclusions*. Strong caspase-3 expression on AMM cells could reflect a cellular attempt at the homeostatic autoregulation of the tumor size. Survivin expression on AMM cells is similar to the survivin expression reported on benign meningiomas. Caspase-3 and survivin expression has no prognostic significance on the survival of patients with AMM.

## 1. Introduction

Malignant meningiomas (MM) account for 1–3% and atypical meningiomas (AM) for about 20% of all meningiomas [1]. Prognostic factors associated with recurrence of atypical and malignant meningiomas (AMM) are not agreed upon. Primary AMM are rather sporadic entities, so only few studies on larger series have been performed so far [2–5]. High mitotic count, brain invasion, and parasagittal location are the strongest predictors of shorter recurrence-free survival (RFS) in patients with primary AMM [5]. 

Apoptosis, a regulated form of cellular self-degradation, plays an important role in embryological development as well as in many pathological processes in adults, including tumorogenesis. While necrosis is a direct consequence of cellular ischemia and is dependent exclusively of external factors, apoptosis is triggered inside the cell. It can be regarded as a cellular response to stimuli not immediately lethal. Anything producing cell necrosis by direct cell destruction can induce apoptosis if the cell initially survives. 

Tumor growth depends on balance between cell gain and cell loss, apoptosis being the most significant component of continuous cell loss in tumors. When tissue proliferation is self-limited by the mechanism of apoptosis, tumor growth is stopped. If balance between cellular proliferation and apoptosis is destroyed, tumor growth continues. Accelerating apoptosis in vitro can slow tumor growth, including meningioma growth [6]. Mistakes in the apoptosis mechanism are present meningioma cells [7, 8]. 

Positive correlation between apoptotic index (AI) and proliferation index (PI) was shown in meningiomas [8]. AI was also found to be a predictor of meningioma recurrence [8].

Apoptosis is controlled by transcriptional and nontranscriptional mechanisms. *Caspases* are cystein proteases playing a key role in nontranscriptional regulation of apoptosis. Central role in this regulation path is played by the *effector caspase-3*. Immunohistochemical labeling of caspase-3 expression can be used to measure level of apoptosis instead of AI. Caspase-3 overexpression was correlated to higher grade of meningioma and was found to be a predictor of recurrence of meningiomas [2, 7]. 

Caspases are regulated by several proteins, among them *inhibitor of apoptosis proteins (IAP). *Survivin, an IAP, blocks apoptosis by binding to effector caspases-3 and caspases-7. It is expressed in the proliferative phase of the cell cycle [9]. Many studies have proved the involvement of survivin into pathogenesis of different kinds of tumors, including tumors of the CNS [9, 10]. Expression of survivin could be an early event in tumorogenesis [11]. Expression of survivin appears to correlate with resistance to radiotherapy in glioblastomas [12]. 

So far, only few studies measuring expression of survivin in meningioma cells have been performed [9, 13, 14]. Attempts were made to correlate survivin expression on meningioma cells with meningioma grade. Survivin was found to be strongly expressed in benign meningiomas (BM) [9, 11, 14]. In one series, higher survivin expression was found in MM compared to BM [13]. No correlation between survivin expression and meningioma grade, meningioma invasion, or clinical course was found in other series [9]. 

86 patients with primary AMM were included in our study. To the best of our knowledge, this is the largest study reporting expression of apoptotic markers on AMM and the first study evaluating prognostic importance of both caspase-3 and survivin expression on AMM. 

## 2. Materials and Methods

### 2.1. Patients

86 patients with primary intracranial AMM meningiomas, treated surgically in years 1990 to 2005 at UMC Ljubljana, Slovenia, were included in the study. Among 86 cases, 76 (88%) were AM and 10 (12%) were MM. Clinical and histopathological data were obtained from clinical and histopathological reports. The following data were obtained: age and sex of the patients, tumor location, extent of resection, tumor grade, brain invasion, and mitotic count. Tumors were grouped into three categories according to their location: convexity, parasagittal, and cranial base meningiomas. Data about overall survival (OS) and recurrence-free survival (RFS) were collected in cooperation with the Cancer Registry of Slovenia. Only radiologically detected, surgically removed, and histologically confirmed tumors were considered as true recurrences. Follow-up period of 7 to 21 years ended in May 2011. 

### 2.2. Immunohistochemistry

From formalin fixed and paraffin embedded tumor tissue, 5 micrometer thick sections were cut for immunohistochemistry on MIB-1 antigen, surviving, and caspase-3. No special criteria for block selection were used. IHC was performed on random representative tumor sections in immunostainer from Ventana according to standard IHC protocols. PI was determined with computer program counting percentage of nuclei stained with MIB-1 antigen. Survivin and caspase-3 expression were assessed in all selected tumors, without reference to previous diagnosis. Caspase-3 and survivin expressions were evaluated as approximate percentage of stained cells. Expression was scored from 1 to 3. Score 1 described mild staining (<1% positive nuclei), score 2 moderate staining (1–10% positive nuclei), and score 3 strong staining (more than 10% positive nuclei).

### 2.3. Statistical Methods

Statistical analysis was performed with SPSS for Windows (SPSS Inc., Chicago, IL). Following factors were assessed to determine prognostic influence on OS and RFS: age and sex of the patient, tumor location, tumor grade of, PI, presence of brain invasion, mitotic count, and caspase-3 and survivin expression. Spearman's correlation test was used to evaluate correlation between variables. Survival analysis was performed using Cox model and survival curves were estimated using Kaplan-Meier the methods. Final multivariate models were obtained using stepwise backward elimination of nonsignificant variables. 

## 3. Results

### 3.1. Caspase-3 Expression

Caspase-3 immunoreactivity was observed in 60 (71%) of primary AMM. In most of the cases, only individual nuclei were stained. In 26 cases (31%), up to 1% of nuclei were stained, in 30 cases (35%) moderate staining was observed, and in 4 cases (5%) more than 10% cell nuclei were stained. Intensity of caspase-3 expression correlated with tumour grade (*P* = 0.004), PI (*P* = 0.019), and mitotic count (*P* = 0.013). No correlation between caspase-3 expression and age, tumour location or brain invasiveness was found. The intensity of caspase-3 expression is shown in Table 1.

### 3.2. Survivin Expression

74 (88%) of tumor cell nuclei expressed survivin, while in 10 tumors (12%) no survivin expression was found. Survivin staining was mild in 24 (28.6%) tumors, moderate in 25 (29.6%) tumors, and strong in 35 (41.7%) tumors. Survivin tended to be more expressed in female patients (*P* = 0.072). There was no correlation between survivin expression and age, brain invasiveness, mitotic count, PI, or caspase-3 expression. Staining was stronger in MM compared to AM, but the difference was not statistically important (*P* = 0.491). The intensity of survivin expression is shown in Table 1. 

### 3.3. Survival Analysis

Patients were followed until death or for a median of 96 months. At time of followup, tumor had recurred in 31 patients (36%), median 31 months after the diagnosis. 37 patients (43%) died, median 45 months after the diagnosis. Neither survivin nor caspase-3 expression appeared to be significant predictors of OS or RFS in patients with AMM. The univariate Cox analyses of the RFS and OS are shown in Table 2. 

## 4. Discussion

In our study, we showed neither caspase-3 nor survivin expression in primary AMM predicts survival of patients with primary AMM. Caspase-3 and survivin expression was both more prominent in MM compared to AM. However, only the difference in caspase-3 expression between the two groups was statistically significant.

Malignant potential of meningiomas can be evaluated using several cytokinetic markers, such as PI or apoptotic index [8]. While PI has no prognostic significance in BM [15], several studies have shown a correlation between PI and prognosis of patients with AMM [5, 16]. Apoptotic index shows correlation with PI and tendency to rise with meningioma grade [8]. Prognostic implication of apoptosis in meningioma recurrence has been proposed [8].

While no overexpression of caspase-3 was observed in BM [17], caspase-3 expression has so far not been evaluated in primary AMM. Increased caspase-3 expression was correlated to higher meningioma grade and shorter RFS of meningioma patients [2, 7]. Increased apoptosis was also correlated to loss of PR on meningioma cells, linked to meningioma malignancy [18]. In our study, correlation between meningioma grade and caspase-3 expression was also proved, caspase-3 expression being higher in MM compared to AM. However, no independent prognostic influence of caspase-3 expression on RFS or OS was noticed. Correlation between PI, mitotic count, and caspase-3 expression probably reflects a homeostatic autoregulation of tumor size [8]. 

Survivin was found in many neoplasms of the central nervous system [9–11, 19]. It is particularly strongly expressed in benign intracranial tumors, such as meningiomas [9, 11, 14, 20]. In a study of different CNS tumors, all meningiomas regardless of grade were intensely positive for survivin [9]. In a study of 90 BM, survivin was expressed in 94% of tumors [14]. Survivin is also strongly expressed on meningioma cultures in vitro [8, 9]. Expression of survivin on meningioma cells is reported to be similar to expression of survivin on normal cap cells [21, 22]. Antibodies against survivin were found in 11.9% of patients with meningiomas, and not in healthy individuals [23]. 

Overexpression of survivin in BM contrasts with reports relating survivin expression to rapid division and poor prognosis [9]. Correlation between meningioma grade and survivin expression was confirmed in only one study where MM expressed significantly more survivin than BM [13]. Other studies showed no correlation between survivin expression and meningioma grade or brain invasion [9, 21]. In our series, survivin immunoreactivity was observed in 88% of cells of AMM. The expression of survivin was similar to that reported on BM [14]. No correlation between survivin expression and meningioma grade can be concluded from our data. 

While immunoreactivity for caspase-3 in our study was confined to the nuclei, immunoreactivity for survivin was more frequently present in the cytoplasm. The same was observed in other series [13, 14]. We found stronger survivin staining in female patients, which might be explained by hormonal influences in meningioma genesis. 

So far, prognostic influence on survival has not yet been explored on a larger series of AMM. According to our results, survivin expression has no prognostic influence on survival of patients with primary AMM. While higher expression of survivin was observed in recurrent compared to nonrecurrent AMM [13], no correlation between survivin expression and recurrence was found in BM [14]. In our series, survivin expression was not correlated to caspase-3 expression, suggesting survivin expression not being dependent on overall level of apoptosis. 

According to our results, survivin expression is not connected to malignant progression of meningiomas. Survivin being expressed in 94% of BM [14], its overexpression could represent an early stage or even the initiating event in meningioma development. Since it has been proposed as a potential target for immunotherapy in meningiomas [19], it should preferably be attacked already in BM, before malignant transformation occurs. 

Externally, apoptosis in meningiomas can be induced by gamma-rays or chemotherapeutic agents, such as cisplatin, hydroxyurea, or fenretinide [6, 20]. Survivin is highly expressed in BM and may be responsible for poor response of BM to standard chemotherapeutic treatment. In our study, high expression of survivin was also demonstrated on AMM, which may explain high radio- and chemoresistance of all three grades of meningiomas. Survivin might be one of the most important defense mechanisms against apoptosis in meningiomas [24]. Irinotecan and nelfinavir were shown to decrease levels of survivin in meningioma cultures resulting in growth-inhibitory effect on meningioma cells [24, 25]. The effect of irinotecan was stronger on AMM cells [25]. Perhaps, the overall level of survivin is slightly lower in AMM compared to BM, AMM consequently being more sensitive to chemo- and radiotherapy than BM. Further studies comparing survivin expression in BM and AMM are necessary to confirm this hypothesis. 

## Figures and Tables

**Table 1:** Antigenic characteristics of 86 AMM.

Parameter	Patients (*n* = 86)	AM (*n* = 76)	MM (*n* = 10)	*P*
	Range, 0–39.2	Range, 0–39.2	Range, 4.5–30.8	
PI (%)	Mean, 9.4	Mean, 8.0	Mean, 19.5	**0.002**
	Median, 6.8	Median, 6.0	Median, 21.3	

PI (>4%)	58 (67.4)	48 (63.2)	10 (100)	**0.019**

	Range, 0–29.2	Range, 0–16.8	Range, 0.4–29.2	
Caspase 3 (% stained nuclei)	Mean, 9.4	Mean, 1.7	Mean, 4.7	**0.004**
	Median, 0.8	Median, 0.4	Median, 1.6	

	Range, 0–66.7	Range, 0–66.7	Range, 3.3–26.7	
Survivin (% stained nuclei)	Mean, 14.0	Mean, 13.7	Mean, 16.2	**0.491**
	Median, 5.7	Median, 4.0	Median, 20.0	

**Table 2:** Univariate survival analysis of 86 AMM.

Variable	*n*	Recurrence free survival			Overall survival		
		HR	95% CI	*P* value	HR	95% CI	*P* value
Sex (male versus female)	86	1.9	0.9−4.0	0.077	1.9	1.0−3.8	0.064
Age (≥55 versus <55)	86	0.9	0.5−1.8	0.776	3.19	1.5−6.7	**0.002**
Location (parasagittal-falcine versus others)	86	2.3	1.1−4.7	**0.021**	1.5	0.8−3.0	0.238
WHO grade (III versus II)	86	2.0	0.8−5.3	0.145	1.7	0.6−4.3	0.293
Mitotic count (continuous)	86	1.08	1.03−1.13	**0.001**	1.03	0.99−1.08	0.158
Brain invasion when assessable (present versus absent)	37	7.2	0.9−55.7	0.059	4.4	1.0−19.2	**0.049**
Ki67 (%)	86	1.00	0.97−1.05	0.701	1.00	0.95−1.03	0.606
Ki67 (>4% versus ≤4%)	86	2.7	1.1−6.7	**0.027**	1.9	0.9−4.0	0.104
Caspase-3 expression	85	1.0	0.5−2.2	0.375	1.1	0.6−2.1	0.812
Survivin expression	85	0.7	0.3−1.7	0.488	1.0	0.5−2.0	0.994
